# Medicine: Science or Art?

**DOI:** 10.4103/0973-1229.27610

**Published:** 2006

**Authors:** S.C. Panda

**Affiliations:** * *Assistant Professor, Department of Community Medicine, V.S.S. Medical College, Burla, Orissa. Also, Editor, Journal of Community Medicine. Email:*sadhucmvss@yahoo.co.in

**Keywords:** *Art of Medicine*, *Science*, *Evidence-based Medicine*, *Human Faculty*, *Humanistic Education*

## Abstract

Debate over the status of medicine as an Art or Science continues. The aim of this paper is to discuss the meaning of Art and Science in terms of medicine, and to find out to what extent they have their roots in the field of medical practice. What is analysed is whether medicine is an “art based on science”; or, the “art of medicine” has lost its sheen (what with the rapid advancements of science in course of time, which has made present day medicine more sophisticated). What is also analysed is whether the “science of medicine” is a pure one, or merely applied science; or the element of science in it is full of uncertainty, simply because what is accepted as “scientific” today is discarded by medical practitioners tomorrow in the light of newer evidence. The paper also briefly touches upon how, in the field of present medical education, the introduction of medical humanities or humanistic education has the potential to swing the pendulum of medicine more towards the lost “art of medicine”.

The paper concludes by saying that the art and science of medicine are complementary. For successful practice, a doctor has to be an artist armed with basic scientific knowledge in medicine.

## Introduction

Medicine is what helps or heals. From time immemorial, man has been struggling to control disease. Medicine has advanced with the progress of science. It is thus built on the best of the past. Park (2002), discussing *Medicine in Antiquity* has rightly quoted Dubos:

Ancient medicine was the mother of science and played a large role in the integration of early culture.

Ancient medicine across the globe was different due to vivid cultures and civilizations. In due course, this was enriched by integration of cultures across many geographical boundaries, races and ethnic groups. Due to this, medicine has undergone wide changes, so much so that its definition itself has metamorphosed many times.

What, then, is medicine? Many people think it is a science, others think it is an art. Another group is of the view that medicine is both an art and a science. Rogers (2006), in his *Introduction to the Study of Medicine*, says:

Medicine is sometimes considered a science, and sometimes an art; the object of medical science is to study disease.

Steve Solomon has tried to define medicine in the first chapter of hygiene library catalog of his website. In his discussion he differs from the view of Rogers quoted above. According to Solomon (2006):

Man should be studied in life and health-the influences on the body of food, clothing, bathing, and the daily care of the body. A live man, well understood, is worth more from a health standpoint than thousands of dead men. The aim of medical art is to restore and maintain health.

He further points out:

Medicine is supposed to be a scientific study and its practice an art. The study of disease requires the aid of science. Consummate art is required to effect a cure when nature is no longer able to help herself.

I have tried to take steps here to unfold the mystery over the status of medicine by an in-depth analysis.

## Is Medicine An Art?

When Decyk (1996) was delivering his presidential address in Leonardo's workshop on *The fine art of teaching philosophy*, he said sometimes people opined that teaching philosophy was an art. In order to explain it, he gave an example of distinguishing the art of medicine from its science cited commonly by people: “Sometimes people say, ′The practice of medicine is an art, not a science′, or, ′The practice of medicine is an art, not an exact science′.” He went ahead by saying that an Art involved “a skill acquired by experience” or observation.

That is applicable as much to the teaching of philosophy, as to the practice of medicine.

Saunders (2000) quotes Thomas Huxley in his paper thus:

Applied science is nothing but the application of pure science to particular classes of problems. No one can safely make these deductions unless he or she has a firm grasp of the principles. Yet the idea of the practice of clinical medicine as an art persists.

What exactly, then, is the *art* of medicine? Hegde (1999) speaks of medicine as an *art based on science*. The art of medicine remains the same and is the strong foundation of practice. It is permanent and has evolved through the centuries based on human values and intuition. Its thrust is to allay anxiety in the minds of patients and to console them under all circumstances. He describes further:

Some years ago, survey done in Thailand showed that all kinds of doctors ranging from the quacks to the best trained modern practitioners have been equally effective in society if they had human qualities of head and heart required to encourage the patients′ own healing power.

As per my observation, even in the villages, quacks, without any scientific training, provide care to the needy. Still people accept them; they are available and kind to the sick. According to Cecil's Textbook of Medicine (Goldman and Dennis, 2004), the art of caring and comfort, guided by millennia of common sense as well as a more recent systematic approach to medical ethics, remains the cornerstone of medicine - without these humanistic qualities the application of modern science of “medicine” is suboptimal, useless, even detrimental.

## Is Medicine A Science?

Unlike physics or chemistry, medicine is not a pure science. When we call it an applied science, it implies only principles of pure science are applied in medicine. Even the results obtained from sophisticated tools may be different. One pathologist may opine about a particular case as malignant, which may not be corroborated if some other colleague examines it. Hegde (1999) has rightly mentioned that scientific truths are not true for all times, unlike truths in the field of the art of medicine in science. Today's truth may be tomorrow's folly. The half-life of truth in medicine is short. There is a saying (Lakshmipati, 2003):

Half of what is true today will be proven to be incorrect in the next five years. Unfortunately we don't know which half that is going to be.

A small example can be discussed here: the formulation of oral rehydration salts. WHO (2002) adopted the new ORS (low sodium, low glucose) formula to fight diarrhoea among under five children. This change became necessary after studies conducted in five developing countries. Similarly, with surgical procedures. Many of them become outdated and surgeons adopt newer procedures to treat various problems. For example, the concept of a ripe cataract is outdated. Today, ophthalmologists opine that cataracts should be removed when they cause symptoms by dissolving and removing the cataractous lens with ultrasound (often referred to as phacoemulsification). A clear, synthetic lens is then put in place (Cataract FAQS, 2006). The surgical procedure called Thiersh operation in treating prolapsed rectum has become obsolete. Delorme's operation is now the preferred operation (William Norman, 2004).

Management of diseases, even diagnostic methods and ideas on causation of a particular disease, also change with passage of time.

## Art Of Medicine?

Warsop (2002) asks in his article, “Is there anything intrinsic to medical practice that can reasonably be called an art?” According to Saunders (2000), the art is not merely part of the “medical humanities” but is integral to medicine as an applied science, which requires what he calls a “doctrine of standard empiricism”. This is described as a mode of inquiry the aim of which is to promote “objective knowledge and truth” and to provide explanations and understanding. Doctors undertake various kinds of activities, which, though not scientific, are essential to the practice of medicine as a science. These sorts of activities, constructed with evidence-based medicine, collectively constitute the art of medicine.

In Cecil's Textbook of Medicine (Goldman and Dennis, 2004), medicine is

…a profession that incorporates science and scientific methods with the art of being a physician. The art of tending to the sick is as old as humanity itself. Compared with its long and generally distinguished history of caring and comforting, the scientific basis of medicine is remarkably recent. Further the physician is advised to understand the patient as a person. Three fundamental principles are important to practitioners. They are primacy of patient welfare, patient autonomy and social justice.

The first principle lays emphasis on the patient. Patient's interest, concern or welfare comes first. The plethora of diagnoses and treatment options are secondary and subsidiary to patient welfare. The second principle speaks of the final decision about his or her treatment option, which lies with the patient. A doctor only recommends. In the process of dealing with patients, social justice again is of utmost priority. It is important because the doctor is responsible for the individual patient and to the society at large. He should ensure that health care and health services are equally accessible and available to people of all strata of society.

In Cecil's Textbook of Medicine (Goldman and Dennis, 2004), art of medicine is conceived of as the activity of patient advocacy by means of human faculty, and the role of science as subordinate to the humane art of listening and advocacy is highlighted. Another view is that the goal of medicine is to produce healing or health for the sake of the patient, and not for the sake of art (Goldman and Dennis, 2004), whereas Saunders (2000) sees the art of medicine as part of the culture of science. Warsop (2002) says:

The cosmetic surgeon takes aesthetic factors into consideration as part of her daily work, but such factors are subsumed by the priority to restore her patient's health.

Warsop means to say that cosmetic surgeon's goal is not to create art, using her patient as raw material, as a potter uses clay. Citing this example, he concludes that medicine fails to qualify as an art in the sense of art understood as fine art, as say painting or sculpture.

## Art Versus Science

On many occasions doctors are criticized, abused and manhandled not because of their paucity of knowledge. Rather, it is related to their insensitive behaviour and for completely ignoring the emotional distress and strain affecting a sick individual. Mahajan (2006) cautions the physician not to allow scientific medicine to blunt his humanity, ignore ethics and the need for empathy. Hegde (1999) is of the view that doctors of all hues and colours have succeeded in practice mainly because they show concern for their patients and become beneficient towards them.

The art of medicine deals with the whole gamut of doctor-patient relationship. Most patients think that high-tech medicine can do wonders for suffering humanity. While it can do a lot in special situations like emergency care, in all other areas, the art of medicine rules the roost. Even in an emergency, human compassion can do a lot to assist the protean machines, which can appear quite frightening to the critically ill. In the outdoor, indoor, operation theatre, labour room, during various investigations or in any survey of the community, everywhere, the doctor-patient relationship requires compassion, a caring attitude from the doctors, besides communication skills (Hegde, 1999). A surgeon, physician or any health care provider, needs to be essentially a good human being. A knife only cuts or a drug assists, along with the availability of best possible technology, drugs and other logistics. The vital forces of the body and the intense desire to live, or the positive attitude of the patient, are what really count. Also, other major factors such as concern, sympathy, compassion, assurance and other humane qualities of the doctor, which can be termed the art of medicine, are of much importance in practicing medicine. Diagnosing disease and choosing the best treatment certainly requires scientific knowledge and technical skills in health care professionals. But only this much won't do. Achtenberg (1996) said:

A medicine that cares or cures, helps or heals has an even greater consequence for humanity than that of merely mending, tending, patching or preventing the various ailments that are the result of being alive.

Practicing the *art* of medicine one can mend the aches and pains of fellow human beings. The act of giving service with a humane touch - in the form of medicine, is the purest gesture of peace and communication; or we can say, manifestation of medicine in an art form (Achtenberg, 1996)

## Medicine Is Both Art And Science

So far we have discussed art of medicine as a human faculty that has to be based on science. Medicine, however, is not an exact science. It is an applied science, and its practice is an art.

Then what exactly is medicine? In effective medicine, the power imbued in the caregiver is based on trust, which may itself be integrated with the healing process. If people have trust and confidence in their provider, they follow their recommendations. If trust is absent they won't. Trust blossoms not only out of competency or skill; it involves sensitivity to another world-view. Moreover, trust evolves out of the persona of the caregiver (Achtenberg, 1996). According to Smith and Taylor (1996):

That Medicine is a science is the popular belief, and this has been reinforced by the advent of ′evidence-based medicine. However, the view of science implied is a narrow one, foreign both to pure scientists and to artists and the art of medicine is devalued by this approach. There are both important differences and important similarities between science and the arts. The arts should contribute to evidence-based practice and education along with science, and have a role in many aspects of medical practice.

Saunders (2000) says, “The practice of clinical medicine with its daily judgments is both science and art. In the practice of clinical medicine, the art is not merely part of the ′medical humanities′ but is integrated to medicine as an applied science”. Warsop (2002) finally agrees by saying that science is of course essential to medicine, but medicine cannot be simply identified with pure science or even with applied science. The art of medicine is essentially composed of the clinical skills of listening and advocacy brought to bear in the consultation.

Tucker (1999) describes medicine as an art as well as a science. He says:

We all know this but over the ages this art/science ratio has undergone a dramatic change. The medical pendulum is swinging from the art to the science side. However, in my opinion, the best clinician is one who armed with this scientific knowledge, practices using excellent clinical judgment (which of course is his art). Compassion and understanding are a large part of this art.

The art of medicine exists since the time of primitive medicine. But the science of medicine changes with the progress of science and changing concepts from time to time. So, the art of medicine, or compassion, care, sympathy etc, are the building blocks of the practice of the science of medicine.

## Revive The Dying Art Of Medicine

Trousseau (1869) says:

The worst man of science is he who is never an artist, and the worst artist is he who is never a man of science. In early times, medicine was an art, which took its place at the side of poetry and painting; today they try to make a science of it, placing it beside mathematics, astronomy, and physics.

He means to say that with progress of science and its application, there is a rapid decline in the so-called human elements of health care providers, which dilutes the age-old doctor-patient relationship.

Philip Overby (2005) says:

Today, doctors are both more powerful and more deaf. They are far less helpless in the face of suffering, yet they often cannot hear the cries that evoke no possibility of remedy. A more humanistic education might heal the physician's deafness. It will not make treating the untreatable any easier, but it may at least leave the doctor less naked on the wards.

Hegde (1999) has also expressed similar concern over the issue. He says:

The art of clinical medicine is dying in the present teaching set up with high-tech gadgets. In the field of medical education these days, there is not much emphasis on the art of medicine. In only one university in the world, at Brisbane, students are recruited into medical schools after they have learnt music, philosophy etc, a very good beginning indeed.

Philip Overby (2005), in his paper, says:

Many writers have argued that art and literature should have a place in the medical curriculum on the grounds that art helps doctors to understand experiences, illness and human values and that art itself can fulfill a therapeutic role. At its best humanistic education will help doctors at the bedside by forcing them to grapple with the kinds of existential question that their patients can avoid.

The importance of medical humanities in medical education is realized across the globe and steps have been taken to introduce it in various medical schools and universities (Evans and Greaves, 2001; Glasser, 2001; Meakin, 2002).

## Conclusion

Medicine is both an art and a science. Both are interdependent and inseparable, just like two sides of a coin. The importance of the *art* of medicine is because we have to deal with a human being, his or her body, mind and soul. To be a good medical practitioner, one has to become a good artist with sufficient scientific knowledge. Technology covered with the layer of art alone can bring relief to the sick.

In the field of medical education, this dying *art* of medicine has to be revived throughout the world. So the conclusion to the debate on the status of medicine as art or science is crystal clear. Let us conclude with the famous words of Albert Einstein (Wikiquote, 2006):

The most beautiful thing we can experience is the mysterious. It is the source of all true art and all science. He to whom his emotion is a stranger, who can no longer pause to wonder and stand rapt in awe, is as good as dead; his eyes are closed.

## Questions That This Paper Raises

1. How to revive the dying art of medicine in this hi-tech age?
2. How do we find out ways and means to integrate the “art of medicine” with the “science of medicine”?
3. What is the present state of medical education in terms of “art of medicine”?
4. What is the role of art of medicine in achieving “health for all” across the globe?
5. Is there a need for change in the medical curriculum so as to produce doctors who will be able to practice medicine to serve humanity with humanistic qualities based on scientific knowledge?

## About the Author

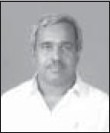



***Sadhu Charan Panda*** *(M.D.) is Editor, Journal of Community Medicine (ISSN 0973-2454). He is also Assistant Professor (Senior Teacher), V.S.S. Medical College, Burla -768017 (Orissa). He was Assistant Surgeon under Orissa Health Service from 17-01-1986 to 19-02-1997 and entered Orissa Medical Education Service from 20-02-1997 and continues till date. He has publications in state and national journals, and had made presentations at various conferences. He has participated in workshops and training programmes in NTI, NICD, Govt. Medical College, Surat, B.H.U. etc. He is a Life member: IPHA, IAPSM, IMA. He is also Member, WAME and Rotary (PHF).*
